# In-hospital psychological stress and post-hospital outcomes

**DOI:** 10.1177/13591053251398884

**Published:** 2026-01-27

**Authors:** Daniel M. Ford, Rebecca Lawton, Elizabeth A. Teale, Daryl B. O’Connor

**Affiliations:** 1 University of Leeds, England, UK; 2 Bradford Institute for Health Research, UK

**Keywords:** patients, stress, hospital, post-hospital syndrome, loneliness

## Abstract

Psychological stress experienced by hospital inpatients has been shown to be associated with poorer post-hospital outcomes. The current study aimed to test for associations between hospital-related stress and post-hospital outcomes, and explore whether these associations varied by stressor type, demographic, and other patient factors. A nationally representative sample of 660 recent UK inpatients completed the Hospital Stress Questionnaire and four post-hospital outcome measures. Increased in-hospital stress was observed amongst patients that were younger, female, from an ethnic minority, had a longer hospital stay, or an unplanned admission. In-hospital stress was associated with all four post-hospital outcome measures. The associations were stronger for subjective than objective outcomes, and strongest with stressors related to health anxiety and negative effects of treatment. This study provides further evidence that in-hospital stress is associated with poorer post-hospital outcomes. Future interventions ought to focus on reducing in-hospital stress to improve patients’ experiences of healthcare and recovery.

## Introduction

Psychological stress has been repeatedly shown to have direct (via psychobiological processes) and indirect (via changes in health behaviours) impacts on health (O’Connor et al., 2021). This is particularly evident during hospitalisation; inpatients are exposed to a large variety of stressors, which have recently been linked to poorer health outcomes (Ford et al., 2023). Hospital-related stressors, such as disrupted sleep, feelings of helplessness, and missing loved ones, can cause psychological and physiological strains. Over the course of the hospital stay, these strains cause a ‘wear and tear’ effect on the body (known as *allostatic overload* (McEwen, 2018), and have been associated with negative health outcomes, such as cardiovascular disease and depression (Guidi et al., 2020). This vulnerability to adverse events can follow patients for weeks after being discharged, and is known as *post-hospital syndrome* (Krumholz, 2013).

Post-hospital syndrome can be costly to both patients and health services, due to an increased rate of unplanned readmissions and emergency department visits (Rawal et al., 2019), among other adverse events (Schattner, 2023). However, these costs may be reduced if exposure to stress during hospitalisation can be reduced (Goldwater et al., 2018). The Hospital Stress Questionnaire (HSQ) was developed and validated with the aim of identifying and measuring these hospital-related stressors (Ford et al., 2024, 2025). The HSQ comprises seven dimensions of in-hospital stress: the patient’s quality of care, being away from home, being inconvenienced, anxiety around health, suffering negative effects of treatment, the ward environment, and having a disrupted patient experience. Taken together, these domains make up a composite score of in-hospital stress, which indicates the stressors experienced by a patient, and the extent to which the patient perceived each one to be stressful.

The HSQ has been shown to have a negative association with health-related quality of life and self-rated health in the 2 weeks post-discharge (Ford et al., 2025) but has yet to be tested for associations with other post-hospital outcomes. Associations between the HSQ and several psychological (e.g. feelings of vulnerability) and physical (e.g. time to return to usual activities) health outcomes would support the theory of allostatic overload being the aetiology behind post-hospital syndrome. Most notably, this would provide evidence to support the significance of the relationship between stress and health within the hospital environment and provide further rationale for policy makers to focus on reducing the stress of hospitalisation for future patients.

However, before these associations can be tested, we must first consider the vast number of factors that may be influencing the complex relationship between stress and health. For example, there is a documented relationship between stress, health, and age (Piazza et al., 2010); it has long been known that stress and coping change with age (Folkman et al., 1987), and age has been shown to be negatively associated with hospital stress (Volicer et al., 1977), but little is known about which hospital-related stressors are more prevalent in younger and older groups. Similarly, sex differences have been noted in the relationship between stress and health (Bale and Epperson, 2015; Weekes et al., 2005) but it is not known how the sexes differ in their experiences of hospital-related stressors. Finally, social isolation has been observed to play a role in gene expression (and therefore health): individuals high in subjective loneliness are more likely to be characterised by increased inflammation and decreased protection from viral illness (Cole et al., 2007; O’Connor et al., 2021).

Therefore, the aims of the current preregistered^1^ study were to test for associations between hospital-related stress and post-hospital outcomes, and to explore whether these associations varied by stressor type, demographic, and other patient factors. The authors hypothesised a small-to-medium size negative association between hospital-related stress and post-hospital outcomes, as was found in a previous systematic review and meta-analysis (Ford et al., 2023).

## Methods

### Design

A cross-sectional online survey was conducted from March to December 2023, as part of a wider validation study (see Ford et al., 2025). To conduct an exploratory factor analysis within the wider study, the research team sought to recruit a sample size of 670 participants (a subject-to-item ratio of 10:1 is recommended, Costello and Osborne, 2005; Nunnally, 1994). The current study received ethical approval from the University of Leeds, School of Psychology Research Ethics Committee (PSYC-737), and was preregistered (AsPredicted #153763). Participants provided written informed consent to conduct the study and for the findings to be published.

### Participants

Six hundred and seventy-two completed responses were received, 12 of which were excluded for not meeting the below criteria, leaving a total of 660 participants. Inclusion criteria were as follows: (i) participants were required to be at least 18 years old, (ii) have stayed in a UK hospital as an inpatient, (iii) in the past 12 months, (iv) for at least 24 hours, and (v) not for paediatric, maternity, or psychiatric care. A consultee was permitted to assist with or complete the survey on behalf of a relative/friend that was unable to participate themselves. Note the sample size was determined for a related study that involved conducting a factor analysis on the HSQ (Ford et al., 2025) and informed by psychometric theory (Costello and Osborne, 2005; Nunnally, 1994), whereby, a subject-to-item ratio of 10:1 is recommended for conducting an exploratory factor analysis, therefore, we aimed to recruit a sample size of around 670 participants.

Participants were recruited from Prolific (www.prolific.com), Care Opinion (www.careopinion.org.uk), the University of Leeds, School of Psychology Successful Ageing Panel, social media, and word of mouth. Those participating via Prolific were compensated with £2 for completion of the study; those recruited via other methods were eligible to be entered into a prize draw to win a £100 gift voucher, or one of three £50 gift vouchers.

As this study recruited primarily online, several measures were taken to identify bots and fraudulent responses – submissions made with fictional data, in an attempt to receive payment for participation (see Silverstein et al., 2024). To identify such responses, screening questions were placed at the beginning of the survey, three attention checks were added within the survey (e.g. ‘Please select ‘7’ to show you are paying attention’), and participants were asked for the name of the hospital at which they were admitted. Should a participant fail any screening or attention question, or name a hospital not based in the UK, that response was excluded.

### Measures

The survey was conducted online and took approximately 15 minutes to complete via Qualtrics software (2023). Questions focused on the participant’s most recent hospital experience, and began with five screening questions to assess the respondent’s eligibility to participate. These were followed by demographic questions such as the participant’s age, sex, ethnicity, level of education, and marital status. The survey then moved onto hospital-related questions: how many times the participant had been in hospital, when their most recent hospital stay was, how long they were in hospital for, which hospital they stayed in, whether or not they had surgery, whether their stay was planned or an emergency. Further questions asked about the participant’s recovery period after leaving hospital, such as: how long it took them to get back to their usual activities (adapted from a question on returning to work (see Petrie et al., 1996), to consider that a large proportion of inpatients are of retirement age), if they experienced any complications (using outcomes seen in the Partners at Care Transitions Measure (PACT-M; Oikonomou et al., 2020); occurrences of sores/wounds not healing, infections, falls, and unplanned contact with a GP or Emergency Department), and how vulnerable they felt (single-item question, scored from 1 to 10). The survey then presented the following questionnaires: the UCLA Loneliness Scale, the Lubben Social Network Scale (LSNS), the HSQ, and the EuroQol 5-Dimension Health Questionnaire (EQ-5D). See Supplemental Materials, Appendix A for the full survey.

#### The hospital stress questionnaire

The HSQ (Ford et al., 2024, 2025) is a patient-reported outcome measure assessing seven domains of inpatient psychological stress. The questionnaire consists of 55 items related to hospital stressors, such as ‘Not sleeping well’, ‘Having pain or discomfort from your treatment’, and ‘Missing loved ones’, which are scored between 1 (no stress) and 10 (extreme stress; see Supplemental Appendix A for the full measure). Medium (28 items) and short (10 items) versions have also been validated. The HSQ-55 has evidence of internal consistency (Cronbach’s alpha for each factor ranges between 0.70 and 0.95), convergent validity (correlating highly with the Perceived Stress Scale (PSS-10), Cohen et al., 1983), known-groups validity (unplanned hospital stays shown to be significantly more stressful than planned stays), predictive validity (negative correlation with health-related quality of life measured by the EQ-5D and EQ VAS, in the 2 weeks post-hospital), and test-retest reliability (*r* = 0.90).

#### EuroQol 5-dimension health questionnaire

The EQ-5D is a widely-used measure for describing and valuing health, composed of five dimensions: Mobility, Self-Care, Usual Activities, Pain/Discomfort, and Anxiety/Depression (Brooks, 1996). Each of these dimensions can be measured using a three-level (EQ-5D-3L) or five-level (EQ-5D-5L) Likert scale (Herdman et al., 2011), both of which have excellent psychometric properties but the five-level version is more sensitive to change (Feng et al., 2021). Using the EuroQol registration, the first author (DF) agreed to the EQ-5D Terms of Use, and no licence agreement was needed.

A participant’s responses to the EQ-5D reveal their *health state* (e.g. 11,111 is indicative of full health); a formula can be applied to this five-digit code to derive an *index value*, which reflects how good or bad a health state is according to the preferences of the general population of a country/region. These preferences are determined using a *value set*; a representative sample from that country/region – the value set for England was used in the current study. A higher index value indicates better health-related quality of life.

#### UCLA loneliness scale

The UCLA-LS is a measure of subjective feelings of loneliness and social isolation, which may be a moderator in the relationship between hospital stress and patient outcomes. Originally a 20-item questionnaire (Russell et al., 1978), a short version of three items has since been validated, exhibiting comparable psychometric properties to the original (Hughes et al., 2004). The three-item version was chosen to avoid unnecessarily burdening the participants. Items ask how often the respondent feels lonely or isolated (the current study focused on the weeks leading up to hospitalisation) and are measured on a three-point Likert scale, from ‘Hardly Ever’ (1) to ‘Often’ (3) with a maximum total score of 9. Higher scores indicate more feelings of loneliness.

#### Lubben social network scale

The LSNS is a measure of social engagement including family and friends, and may also be a moderator in the relationship between hospital stress and patient outcomes. A 12-item and six-item version are available, both with acceptable psychometric properties (Lubben, 1988; Lubben et al., 2006). The six-item version was chosen to avoid unnecessarily burdening the participants. Items asked about the number of friends or relatives the respondent has had contact with in the past month (the current study focused on the month prior to hospitalisation), with six response options: 0, 1, 2, 3–4, 5–8, 9+. Higher scores indicate more social support.

### Analysis

Data was analysed using R Statistical Software (v4.3.2; R Core Team, 2023; data and code can be accessed at: https://github.com/DMFord97/HSQ-associations). Kolmogorov-Smirnov test was used to test for normality. Independent Samples t-tests were employed to compare mean differences in HSQ scores between demographic groups, and Spearman’s rank correlations were used to test for unadjusted associations between the HSQ and other variables. Four hierarchical linear regression sequences were performed to test for associations between the HSQ and four post-hospital outcomes: feelings of vulnerability, time to return to usual activities, health-related quality of life (measured with EQ-5D), and outcomes seen in the PACT-M (sores/wounds not healing, infections, falls, and unplanned contact with GP/A&E). For each regression model, age, sex, and ethnicity (white vs other ethnic groups) were controlled for in step 1, the HSQ-55 total score was entered at step 2, the UCLA-LS and LSNS were entered as moderators at step 3, and interaction terms were entered at step 4. This process was repeated using the HSQ-55 subscales to further explore the relationships; however, interaction terms were not entered for each subscale to reduce the number of comparisons. All continuous variables were mean centred before entering into the regression analyses, and standardised beta values were used to allow comparisons. To account for the large number of regression analyses, a more conservative threshold for significance of *p* = 0.01 was employed for these tests (e.g. see O’Connor et al., 2025).

## Results

### Descriptive statistics

Of the 660 participants, 366 were female (55.5%), 292 were male (44.5%), and 2 selected either ‘other’ or ‘prefer not to say’ (0.1%). Ages within the recruited sample ranged from 18 to 97 years (*M* = 54.0, SD = 17.3), and were diverse in ethnicity, education, and marital status. The average length of stay (*M* = 4.73 days; range: 1–180 days) was comparable to the NHS national average of 4.8 days (NHS Digital, 2023). See Ford et al. (2025) for a more detailed description of the sample and comparisons to national inpatient statistics. HSQ total scores ranged from 55 to 513 out of a maximum 550, with a mean score of 208.4 (SD = 92.9), UCLA Loneliness Scale scores ranged from 3 to 9 (*M* = 4.3, SD = 1.8), and LSNS scores ranged from 6 to 36 (*M* = 20.3, SD = 6.1).

### Associations with in-hospital stress

Comparison of mean stress levels revealed significant differences in HSQ-55 total scores for sex, ethnicity, planned/unplanned hospital stays, and surgical/medical patients, but not for education or marital status (see Table 1). Participants that were male, white, had planned to stay in hospital, or were receiving surgery, reported less in-hospital stress than their counterparts. While the majority of surgical patients had planned on staying in hospital (181/276, 66%), likely for elective surgery; very few medical patients had planned on staying in hospital (40/380, 11%), and were likely admitted for emergency treatment. After controlling for planned/unplanned hospital stays, via an ANCOVA, the difference in HSQ-55 total scores between medical and surgical patients was no longer significant (*t* = *–*0.30, *p* = 0.768).

**Table 1.** Differences in HSQ total scores between variables.

Demographic	HSQ-55 total score		Independent samples *t*-test
Sex	Male (*n* = 292), *M* = 193.9	Female (*n* = 366), *M* = 220.1	*t* (631.5) = *–*3.64, *p* < 0.001
Ethnicity	White (*n* = 549), *M* = 204.0	Other ethnic groups (*n* = 111), *M* = 230.6	*t* (164.6) = *–*2.89, *p* = 0.004
Education	No degree (*n* = 305), *M* = 204.9	Degree (*n* = 355), *M* = 211.3	*t* (614.9) = *–*0.872, *p* = 0.384
Marital status	Married (*n* = 383), *M* = 208.4	Unmarried (*n* = 273), *M* = 207.9	*t* (571.6) = 0.07, *p* = 0.945
Planned stay	Yes (*n* = 223), *M* = 179.1	No (*n* = 437), *M* = 223.3	*t* (438.3) = *–*5.89, *p* < 0.001
Surgery^a^	Yes (*n* = 276), *M* = 191.6	No (*n* = 380), *M* = 219.7	*t* (578.1) = –3.84, *p* < 0.001

*M*: mean.

a When controlling for whether the hospital stay was planned or unplanned, there is no longer a significant difference in HSQ total scores between surgical and medical patients.

Kolmogorov-Smirnov test revealed that the data were not normally distributed (*p* < 0.001). Spearman’s rank correlations were performed, revealing that age, loneliness (UCLA-LS), social support (LSNS), and length of stay, each had a small-to-medium size association with HSQ-55 total scores (see Table 2). Higher levels of in-hospital stress were associated with younger age, higher loneliness, less social engagement and longer hospital stays. The number of previous hospital stays had no association with in-hospital stress.

**Table 2.** Association between HSQ total score and other variables (*N* = 660).

Variable	Spearman’s correlation
Age	*r* = –0.25, *p* < 0.001
UCLA-LS	*r* = 0.35, *p* < 0.001
LSNS	*r* = –0.18, *p* < 0.001
Number of stays in lifetime	*r* = –0.00, *p* = 0.989
Number of stays in past year	*r* = –0.01, *p* = 0.755
Length of stay	*r* = 0.09, *p* = 0.017

UCLA-LS: UCLA loneliness scale; LSNS: Lubben social network scale.

To further explore demographic differences, *t*-tests and correlations were employed to observe how sex, age, and ethnicity differed amongst the seven HSQ factors (see Supplemental Materials, Appendix B). Females were significantly more stressed than males in all factors except for the Inconvenienced factor (#3), where there was no significant difference between sexes. For all seven factors, there was a significant, small-to-medium sized negative association with age, implying that in-hospital stress decreases with age. To explore ethnic differences, white participants were compared against other ethnicities (Asian, black, mixed, and other); where other ethnic groups were significantly more stressed in three of the seven factors (#2 Away from home, #3 Inconvenienced, and #7 Disrupted patient experience).

### In-hospital stress and post-hospital outcomes

**Figure 1.**
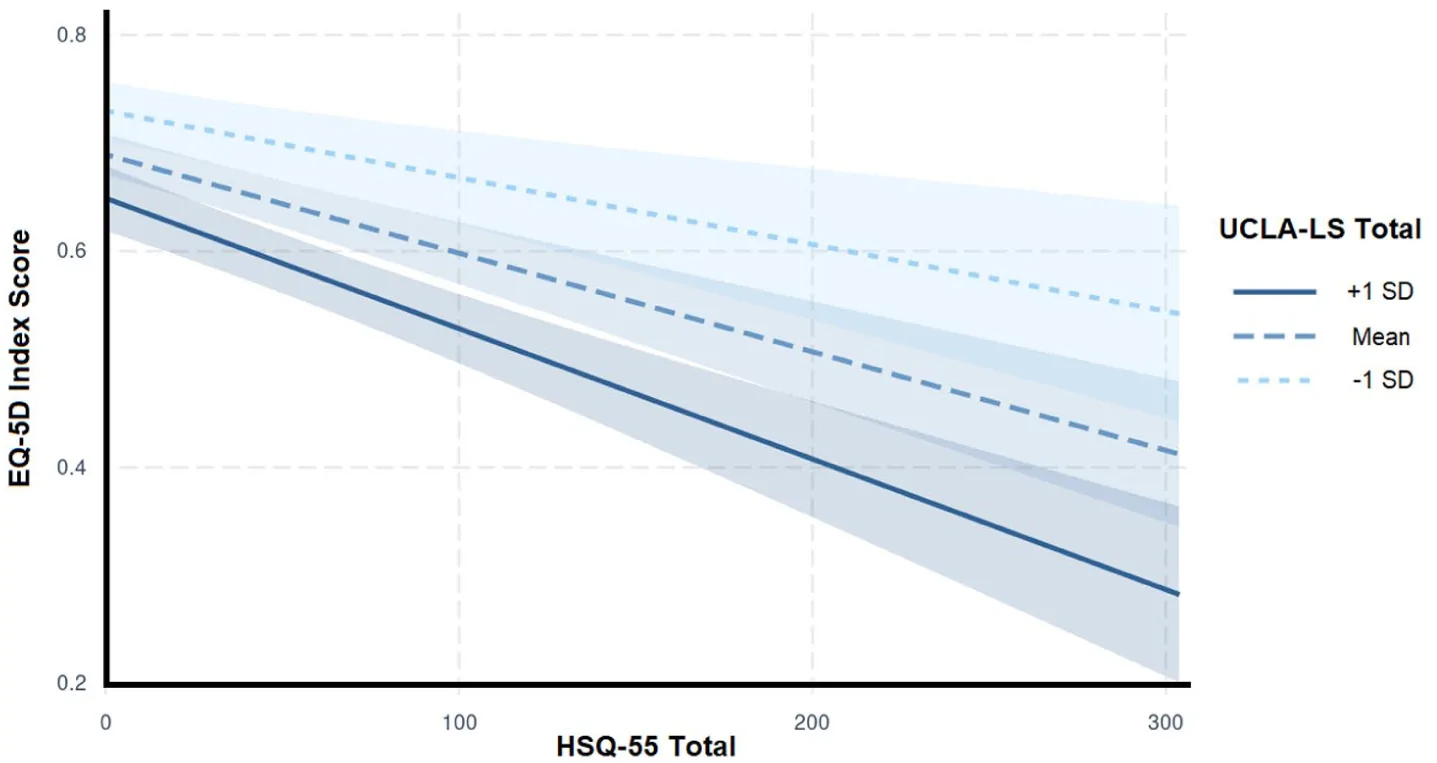
Simple slopes plot depicting the interaction between subjective loneliness (UCLA-LS) and perceived in-hospital stress (HSQ-55) on health-related quality of life (EQ-5D).

Hierarchical regressions were conducted to test whether the HSQ was associated with post-hospital outcomes (see Table 3). At step 1, age significantly entered three of the four models, explaining between 1.6% and 6.6% of variance. Sex and ethnicity did not enter any of the models at the aforementioned threshold of *p* = 0.01. At step 2, the HSQ significantly entered all four models, explaining an additional 1.8%–13.8% of variance. At step 3, feelings of loneliness (captured by the UCLA-LS) significantly entered all four models, and social support (captured by the LSNS) significantly entered one model, together explaining an additional 1.2%–3.0% of variance. At step 4, the interaction term reached statistical significance in one model, explaining and additional 0.9% of variance. The interaction was explored using simple slopes analysis, and as shown in Figure 1, the negative relationship between in-hospital stress and health-related quality of life was stronger at higher levels of subjective loneliness (steeper slope in Figure 1) compared to lower levels (more gentle slope in Figure 1).

**Table 3.** Hierarchical regression analyses testing the effects of in-hospital stress on post-hospital outcomes (*N* = 660).

		β step 1	β step 2	β step 3	β step 4	Δ*R*^2^ for step	Total *R*^2^	*F*-change
Post-hospital feelings of vulnerability								
Step 1	Age	0.123**	0.205***	0.213***	0.214***	0.017	0.017	3.60*
	Sex	0.057	0.010	0.006	0.005			
	Ethnicity	0.008	−0.002	−0.009	−0.008			
Step 2	HSQ-55		0.386***	0.349***	0.349***	0.138	0.155	104.42***
Step 3	UCLA-LS			0.121**	0.124**	0.012	0.167	4.42*
	LSNS			0.020	0.021			
Step 4	HSQ-55 × UCLA-LS				–0.015	0.001	0.167	0.10
	HSQ-55 × LSNS				–0.018			
Time to return to usual activities								
Step 1	Age	0.234***	0.264***	0.280***	0.273***	0.066	0.066	15.04***
	Sex	0.010	–0.008	–0.015	–0.013			
	Ethnicity	−0.050	−0.054	−0.059	−0.060			
Step 2	HSQ-55		0.143***	0.101*	0.097*	0.019	0.085	13.26***
Step 3	UCLA-LS			0.171***	0.144**	0.024	0.109	8.45***
	LSNS			0.085*	0.086*			
Step 4	HSQ-55 × UCLA-LS				0.066	0.005	0.114	1.97
	HSQ-55 × LSNS				–0.023			
EQ-5D								
Step 1	Age	–0.112**	–0.194***	–0.211***	–0.202***	0.020	0.020	4.32**
	Sex	–0.087*	–0.039	–0.032	–0.036			
	Ethnicity	0.012	0.022	0.029	0.035			
Step 2	HSQ-55		–0.386***	–0.336***	–0.331***	0.139	0.158	105.03***
Step 3	UCLA-LS			–0.196***	–0.158***	0.030	0.189	11.85***
	LSNS			–0.083*	–0.082*			
Step 4	HSQ-55 × UCLA-LS				–0.120**	0.010	0.198	3.86*
	HSQ-55 × LSNS				–0.042			
Outcomes seen in PACT-M (sore/wound healing, infections, falls, contact GP/A&E)								
Step 1	Age	0.100*	0.144***	0.163***	0.161***	0.011	0.011	2.29
	Sex	0.039	0.013	0.004	0.005			
	Ethnicity	0.004	−0.002	−0.003	−0.005			
Step 2	HSQ-55		0.207***	0.173***	0.172***	0.040	0.050	26.71***
Step 3	UCLA-LS			0.175***	0.164***	0.030	0.080	10.39***
	LSNS			0.135**	0.135**			
Step 4	HSQ-55 × UCLA-LS				0.032	0.001	0.080	0.25
	HSQ-55 × LSNS				0.007			

* *p* < 0.05, **p < 0.01, ***p < 0.001.

To further explore relationships, the hierarchical regressions were repeated using the seven subscales of the HSQ, rather than its total score (see Table 4 for a summary of the main findings, Supplemental Materials, Appendix C for the full regression findings, and the HSQ in Supplemental Appendix A for the items that comprise each subscale). For post-hospital feelings of vulnerability, at step 2, 24.8% of the variance was accounted for, but only factors #4 (Health anxiety) and #5 (Negative effects of treatment) significantly entered the model. As health anxiety and negative effects of treatment increased, so did post-hospital feelings of vulnerability.

**Table 4.** Main findings of the hierarchical regression analyses testing the effects of the HSQ subscales on post-hospital outcomes (*N* = 660).

	Standardised β values (from Step 2)			
HSQ subscales	Vulnerability^a^	Activities^b^	EQ-5D^c^	PACT-M^d^
Factor 1. Quality of care	0.101	0.124	–0.200**	0.160*
Factor 2. Away from home	–0.013	–0.068	0.072	–0.053
Factor 3. Inconvenienced	–0.101	–0.099	0.063	–0.166**
Factor 4. Health anxiety	0.388***	0.193**	–0.279***	0.263***
Factor 5. Negative effects of treatment	0.243***	0.119*	–0.446***	0.111*
Factor 6. Ward environment	–0.107	–0.063	0.127*	–0.033
Factor 7. Disrupted patient experience	–0.046	–0.045	0.196***	–0.048
Variance explained by HSQ factors (*R*^2^)	0.248	0.057	0.312	0.100

a Post-hospital feelings of vulnerability.

b Time to return to usual activities.

c Health-related quality of life (EQ-5D index score).

d Outcomes seen in PACT-M (sore/wound healing, infections, falls, contact GP/A&E).

* *p* < 0.05, **p < 0.01, ***p < 0.001.

For time to return to usual activities, at step 2, only factor #4 (Health anxiety) significantly entered the model, and 5.7% of the variance was explained, such that higher levels of health anxiety were associated with more time taken to return to usual activities.

For the EQ-5D, at step 2, four factors significantly entered the model (1. Quality of care, 4. Health anxiety, 5. Negative effects of treatment, and 7. Disrupted patient experience), and 31.2% of the variance was explained. Higher scores on factors #1, #4, and #5 were associated with lower health-related quality of life. Unexpectedly, higher levels of stress reported in factor #7 was associated with an increased quality of life; however, when observing the simple correlation, this was shown to be a suppressor effect (*r* = –0.17, *p* < 0.001).

For the PACT-M outcomes, factors #3 (Inconvenienced) and #4 (Health anxiety) were significant, explaining 10.0% of the variance. Higher health anxiety was associated with increased adverse events. Unexpectedly, more reports of hospital-related inconveniences was associated with fewer post-hospital adverse events; however, the simple correlation showed this to be a suppressor effect (*r* = 0.04, *p* = 0.324).

The hierarchical regressions were once again repeated, comparing the amount of variance explained using the long (HSQ-55), medium (HSQ-28), and short (HSQ-10) versions of the measure (see Supplemental Materials, Appendix D). The differences between the long and medium versions of the measure were negligible, but the amount of variance explained was notably less when using the short version.

## Discussion

The current study examined demographic differences of in-hospital stress, and its associations with post-hospital outcomes. This was accomplished via a retrospective survey, completed by a diverse and representative sample of 660 recent inpatients. It was found that perceptions of in-hospital stress differed by age, sex, ethnicity, length of stay, planned/emergency admissions, and levels of social support and loneliness. Additionally, results showed that the HSQ was significantly associated with poorer post-hospital outcomes; namely, feelings of vulnerability, time to return to usual activities, health-related quality of life, as well as outcomes seen in other hospital surveys, such as the PACT-M (falls, sore/wound healing, infections, and unplanned contact with a GP or Emergency Department). The relationship between in-hospital stress and health-related quality of life was stronger when patients reported feelings of loneliness.

### Demographic differences of in-hospital stress

Certain groups of patients exhibited higher levels of perceived in-hospital stress. Younger patients were more stressed than their older peers; interestingly, this was not due to a lack of familiarity with the hospital environment, as it was found that the number of previous stays had no association with in-hospital stress. The relationship with age was consistent across all seven domains of the HSQ, implying that the stressors reported did not differ by age, but that younger patients found being in hospital as more stressful overall. This finding is consistent with those of Volicer et al. (1977) who also observed a negative association between age and in-hospital stress. Also in accordance with the current study, Volicer found that females reported more in-hospital stress than males (Volicer and Burns, 1977). Results from the HSQ show that this finding was observed across six of the seven domains, which implies that females experience the in-hospital environment to be more stressful. This may be due to a number of factors; for example, males may be less willing to admit feelings of stress (see Matud, 2004), or males and females may be affected by stress differently (Brivio et al., 2020; Volicer and Burns, 1977).

In addition to age and sex, ethnicity was also found to be associated with in-hospital stress. Overall, White patients were significantly less stressed than patients of Asian, black, mixed, and other ethnic backgrounds. This is particularly concerning as the negative impacts linked with the stress of hospitalisation are likely to be exacerbated by other health inequalities that these groups experience, such as the increased risk of adverse events and safety events (Chauhan et al., 2020). Patients from ethnic minority backgrounds are disproportionately burdened by social determinants of health, such as education and social inclusion, which can often be met with unsuitable healthcare (Matthew, 2018). In particular, the current study highlighted three areas for improvement, relating to the three HSQ factors that differed between ethnic groups: being away from home, being inconvenienced, and having a disrupted patient experience. Tackling these factors is likely to require a multi-faceted intervention that addresses racism, the different cultural needs of patients, and where relevant, language barriers.

### In-hospital stress and post-hospital outcomes

The HSQ was significantly associated with all four post-hospital outcomes administered in the current study. The effect was stronger for subjective outcomes (medium-sized effect for health-related quality of life and feelings of vulnerability), and weaker for objective outcomes (small effect size for time to return to usual activities and the PACT-M outcomes). This finding is similar to a recent meta-analysis, which found a small association between in-hospital stress and objective patient outcomes, and a medium effect with subjective patient outcomes (Ford et al., 2023). The size of these associations with the HSQ-55 were matched by the HSQ-28, meaning the medium-length version may be more appropriate for some study designs – for example, if the researcher sought to measure the effects of in-hospital stress but was less interested in identifying all of the individual stressors at play. The HSQ-10 was not as strongly associated with post-hospital outcomes but still appears to be an effective tool, especially for patient groups that would experience the longer versions to be too burdensome.

The time periods considered by three of the four post-hospital outcomes were between 2 and 6 weeks after discharge (the fourth outcome, time to return to usual activities, allowed participants to select from a range of time periods). The time frame of up to 6 weeks was chosen as the effects of post-hospital syndrome have been shown to last up to 45 days (approximately 6.5 weeks) for those patients who neither die nor are readmitted (Dharmarajan et al., 2015), such as those recruited in the current study. The findings that in-hospital stress is associated with a range of outcomes in this time period is consistent with the theory of hospitalisation-induced allostatic overload being the aetiology behind post-hospital syndrome (Goldwater et al., 2018; Krumholz, 2013) – whereby the stress of hospitalisation is thought to have a physiological wear and tear effect, leading the body into a state of vulnerability and increasing the likelihood of adverse events. This is further supported by the current study in that the large majority of participants reported times to return to usual activities of ‘4–6 weeks’ or less.

The HSQ domain with the largest associations across the post-hospital outcomes was health anxiety (measured using a combination of HSQ items regarding health uncertainties), which was significantly related to all four outcomes, followed by negative effects of treatment, which were related to two outcomes. These two health-related factors were the only ones associated with post-hospital feelings of vulnerability and time to return to usual activities. It might be the case that these factors are indirectly measuring the severity of the respondent’s condition (the more serious the treatment, the more likely the patient is to fear for their health), and that these individuals feel more vulnerable and take longer to recover than patients in better health receiving more minor treatments (e.g. Zhang et al., 2016). For health-related quality of life, a more diverse range of stressors were identified as important, including quality of care and disrupted patient experience, as well as the two factors mentioned above. Health anxiety was the only factor associated with the occurrence of falls, sores/wounds not healing, infections, and unplanned contact with a GP or Emergency Department. It is unsurprising that the two health-related factors were associated with the largely health-related outcomes included in the current study; improvement efforts would likely be best targeted in aiming to reduce inpatient anxiety regarding their health (e.g. via education; Weisfeld et al., 2021).

Chiefly, our results suggest that the inpatient experience, regardless of stressors relating to having worse physical health, may have a lasting effect on quality of life and risk of adverse events, for several weeks after discharge. Findings from the current study are in agreement with Krumholz’s work, proposing that hospitalisation is a traumatic event (Detsky and Krumholz, 2014) which leads to a generalised vulnerability to adverse events (Krumholz, 2013). We also found that this relationship can be compounded by loneliness and, to a lesser extent, a lack of social support. Psychosocial factors, such as stress and loneliness, are critical to health (O’Connor et al., 2021), not least in a hospital context. Future interventions might focus on those stressors identified as the most stressful elements of hospitalisation, for example disrupted sleep (Ford et al., 2025), in order to improve patient wellbeing and save resources.

### Limitations

Several limitations were apparent within the current study. The majority of the cohort were recruited via Prolific; a research participant platform which may limit the generalisability of the findings. In addition, given range of recruitment strategies utilised, we were unable to calculate a response rate. Moreover, the current sample was more educated than the general public, we did not collect data on potentially confounding variables such as severity of illness, and was lacking in participants aged over 80 years, a population which made up approximately 20% of NHS inpatients in 2022–23 (NHS Digital, 2023). Age is a significant risk factor for poorer post-hospital outcomes (Schattner, 2023). The lack of representation of participants in age groups at higher risk of poorer outcomes may have lessened the magnitude of the current findings. This limitation necessitates future large-scale study of hospital-related stress and outcomes in older patients.

Secondly, the retrospective nature of the current study may have influenced the accuracy of the results, as it relies on the participant’s ability to recall events (and how they felt about those events) from up to 1 year ago. Additionally, two of the outcomes used were single-item measures with good face validity; however, future work should endeavour to use more detailed measures and further test their validity. Finally, we recognise that the cross-sectional nature of the design prevents any causal relationships from being confirmed. Therefore, the authors suggest that future research addresses some of these limitations by employing the HSQ in hospitals to current inpatients, and adopting a longitudinal design to better assess the predictive ability of the measure with a battery of health outcomes.

## Conclusion

In-hospital psychological stress, measured by the HSQ, is associated with a range of post-hospital outcomes. The relationships were stronger for subjective outcomes than objective, and health-related stressors (health anxiety and negative effects of treatment) exhibited the strongest associations. Patients that were female, younger, from an ethnic minority background, did not plan on staying in hospital, or had a longer length of stay, scored higher on the HSQ. Loneliness and social support also affected in-hospital stress and its complex relationship with post-hospital outcomes.

## Supplemental Material

sj-docx-1-hpq-10.1177_13591053251398884 – Supplemental material for In-hospital psychological stress and post-hospital outcomes Supplemental material, sj-docx-1-hpq-10.1177_13591053251398884 for In-hospital psychological stress and post-hospital outcomes by Daniel M. Ford, Rebecca Lawton, Elizabeth A. Teale and Daryl B. O’Connor in Journal of Health Psychology

## Notes

1. This study and analysis plan were preregistered at AsPredicted (#153763).

## References

[bibr1-13591053251398884] Bale TL Epperson CN (2015) Sex differences and stress across the lifespan. Nature Neuroscience 18(10): 1413–1420. 26404716 10.1038/nn.4112 PMC4620712

[bibr2-13591053251398884] Brivio E Lopez JP Chen A (2020) Sex differences: Transcriptional signatures of stress exposure in male and female brains. Genes, Brain, and Behavior 19(3): e12643. 10.1111/gbb.12643 31989757

[bibr3-13591053251398884] Brooks R (1996) EuroQol: The current state of play. Health Policy 37(1): 53–72. 10158943 10.1016/0168-8510(96)00822-6

[bibr4-13591053251398884] Chauhan A Walton M Manias E , et al. (2020) The safety of health care for ethnic minority patients: A systematic review. International Journal for Equity in Health 19: 118–125. 32641040 10.1186/s12939-020-01223-2 PMC7346414

[bibr5-13591053251398884] Cohen S Kamarck T Mermelstein R (1983) A global measure of perceived stress. Journal of Health and Social Behavior 24: 385–396. 6668417

[bibr6-13591053251398884] Cole SW Hawkley LC Arevalo JM , et al. (2007) Social regulation of gene expression in human leukocytes. Genome Biology 8: 1–13. 10.1186/gb-2007-8-9-r189 PMC2375027 17854483

[bibr7-13591053251398884] Costello AB Osborne J (2005) Best practices in exploratory factor analysis: Four recommendations for getting the most from your analysis. Practical assessment, research, and evaluation 10(1): 7.

[bibr8-13591053251398884] Detsky AS Krumholz HM (2014) Reducing the trauma of hospitalization. Journal of the American Medical Association 311(21): 2169–2170. 24788549 10.1001/jama.2014.3695

[bibr9-13591053251398884] Dharmarajan K Hsieh AF Kulkarni VT , et al. (2015) Trajectories of risk after hospitalization for heart failure, acute myocardial infarction, or pneumonia: retrospective cohort study. BMJ 350: h411. 10.1136/bmj.h411 PMC4353309 25656852

[bibr10-13591053251398884] Feng YS Kohlmann T Janssen MF , et al. (2021) Psychometric properties of the EQ-5D-5L: A systematic review of the literature. Quality of Life Research 30: 647–673. 33284428 10.1007/s11136-020-02688-y PMC7952346

[bibr11-13591053251398884] Folkman S Lazarus RS Pimley S , et al. (1987) Age differences in stress and coping processes. Psychology and Aging 2(2): 171–184. 3268206 10.1037//0882-7974.2.2.171

[bibr12-13591053251398884] Ford DM Budworth L Lawton R , et al. (2023) In-hospital stress and patient outcomes: A systematic review and meta-analysis. PLoS One 18(3): e0282789. 10.1371/journal.pone.0282789 PMC9997980 36893099

[bibr13-13591053251398884] Ford DM Lawton R Teale EA , et al. (2025) Psychometric validation of the Hospital Stress Questionnaire. PLoS One 20(4): e0321188. 10.1371/journal.pone.0321188 PMC11981188 40202959

[bibr14-13591053251398884] Ford DM Lawton R Travis E , et al. (2024) Development and initial validation of a hospital stress questionnaire. Health Psychology and Behavioral Medicine 12(1): 2396135. 39219596 10.1080/21642850.2024.2396135 PMC11363734

[bibr15-13591053251398884] Goldwater DS Dharmarajan K McEwan BS , et al. (2018) Is posthospital syndrome a result of hospitalization-induced allostatic overload? Journal of Hospital Medicine 13(5). 10.12788/jhm.2986 29813141

[bibr16-13591053251398884] Guidi J Lucente M Sonino N , et al. (2020) Allostatic load and its impact on health: a systematic review. Psychotherapy and Psychosomatics 90(1): 11–27. 32799204 10.1159/000510696

[bibr17-13591053251398884] Herdman M Gudex C Lloyd A , et al. (2011) Development and preliminary testing of the new five-level version of EQ-5D (EQ-5D-5L). Quality of Life Research 20: 1727–1736. 21479777 10.1007/s11136-011-9903-x PMC3220807

[bibr18-13591053251398884] Hughes ME Waite LJ Hawkley LC , et al. (2004) A short scale for measuring loneliness in large surveys: Results from two population-based studies. Research on Aging 26(6): 655–672. 18504506 10.1177/0164027504268574 PMC2394670

[bibr19-13591053251398884] Krumholz HM (2013) Post-hospital syndrome – A condition of generalized risk. New England Journal of Medicine 368(2): 100–102. 23301730 10.1056/NEJMp1212324 PMC3688067

[bibr20-13591053251398884] Lubben J Blozik E Gillmann G , et al. (2006) Performance of an abbreviated version of the Lubben Social Network scale among three European community-dwelling older adult populations. Gerontologist 46(4): 503–513. 16921004 10.1093/geront/46.4.503

[bibr21-13591053251398884] Lubben JE (1988) Assessing social networks among elderly populations. Family & Community Health 11(3): 42–52.

[bibr22-13591053251398884] Matthew DB (2018) Just Medicine: A Cure for Racial Inequality in American Health Care. NYU Press.

[bibr23-13591053251398884] Matud MP (2004) Gender differences in stress and coping styles. Personality and Individual Differences 37(7): 1401–1415.

[bibr24-13591053251398884] McEwen BS (2018) Redefining neuroendocrinology: Epigenetics of brain-body communication over the life course. Frontiers in Neuroendocrinology 49: 8–30. 29132949 10.1016/j.yfrne.2017.11.001

[bibr25-13591053251398884] NHS Digital (2023) Hospital Admitted Patient Care and Adult Critical Care Activity 2022-23. Available at: https://digital.nhs.uk/data-and-information/publications/statistical/hospital-admitted-patient-care-activity/2022-23

[bibr26-13591053251398884] Nunnally JC (1994) Psychometric Theory. 3rd edn. New York: McGraw-Hill.

[bibr27-13591053251398884] Oikonomou E Page B Lawton R , et al. (2020) Validation of the partners at Care Transitions Measure (PACT-M): Assessing the quality and safety of care transitions for older people in the UK. BMC Health Services Research 20(1): 608. 32611336 10.1186/s12913-020-05369-1 PMC7329420

[bibr28-13591053251398884] O’Connor DB Branley-Bell D Green JA , et al. (2025) Effects of childhood trauma on sleep quality and stress-related variables in adulthood: Evidence from two multilevel studies. Psychology and Health 40: 975–996. 37975565 10.1080/08870446.2023.2281712

[bibr29-13591053251398884] O’Connor DB Thayer JF Vedhara K (2021) Stress and health: A review of psychobiological processes. Annual Review of Psychology 72: 663–688. 10.1146/annurev-psych-062520-122331 32886587

[bibr30-13591053251398884] Petrie K Weinman J Sharpe N , et al. (1996) Role of patients’ view of their illness in predicting return to work and functioning after myocardial infarction: Longitudinal study. British Medical Journal 312: 1191–1194. 8634561 10.1136/bmj.312.7040.1191 PMC2350970

[bibr31-13591053251398884] Piazza JR Almeida DM Dmitrieva NO , et al. (2010) Frontiers in the use of biomarkers of health in research on stress and aging. Journals of Gerontology. Series B, Psychological Sciences and Social Sciences 65(5): 513–525. 20647348 10.1093/geronb/gbq049 PMC2920946

[bibr32-13591053251398884] Rawal S Kwan JL Razak F , et al. (2019) Association of the trauma of hospitalization with 30-day readmission or emergency department visit. JAMA Internal Medicine 179(1): 38–45. 30508018 10.1001/jamainternmed.2018.5100 PMC6583419

[bibr33-13591053251398884] R Core Team (2023) R: A Language and Environment for Statistical Computing. R Foundation for Statistical Computing. Available at: https://www.R-project.org/

[bibr34-13591053251398884] Russell D Peplau LA Ferguson ML (1978) Developing a measure of loneliness. Journal of Personality Assessment 42(3): 290–294. 660402 10.1207/s15327752jpa4203_11

[bibr35-13591053251398884] Schattner A (2023) The spectrum of hospitalization-associated harm in the elderly. European Journal of Internal Medicine 115: 29–33. 37391309 10.1016/j.ejim.2023.05.025

[bibr36-13591053251398884] Silverstein P Pennington CR Branney P , et al. (2024) A registered report survey of Open Research Practices in psychology departments in the UK and Ireland. British Journal of Psychology 115: 497–534. 38520079 10.1111/bjop.12700

[bibr37-13591053251398884] Volicer BJ Burns MW (1977) Preexisting correlates of hospital stress. Nursing Research 26(6): 408–415. 243792 10.1097/00006199-197711000-00009

[bibr38-13591053251398884] Volicer BJ Isenberg MA Burns MW (1977) Medical-surgical differences in hospital stress factors. Journal of Human Stress 3(2): 3–13. 864251 10.1080/0097840X.1977.9936082

[bibr39-13591053251398884] Weekes NY MacLean J Berger DE (2005) Sex, stress, and health: Does stress predict health symptoms differently for the two sexes? Stress and Health 21(3): 147–156.

[bibr40-13591053251398884] Weisfeld CC Turner JA Dunleavy K , et al. (2021) Dealing with anxious patients: A systematic review of the literature on nonpharmaceutical interventions to reduce anxiety in patients undergoing medical or dental procedures. Journal of Alternative and Complementary Medicine 27(9): 717–726. 34076531 10.1089/acm.2020.0504

[bibr41-13591053251398884] Zhang M Hong L Zhang T , et al. (2016) Illness perceptions and stress: Mediators between disease severity and psychological well-being and quality of life among patients with Crohn’s disease. Patient Preference and Adherence 10: 2387–2396. 27920505 10.2147/PPA.S118413 PMC5125764

